# Enhancer-driven transcription of MCM8 by E2F4 promotes ATR pathway activation and glioma stem cell characteristics

**DOI:** 10.1186/s41065-023-00292-x

**Published:** 2023-06-22

**Authors:** Yu-meng Sun, Yi-meng Zhang, Hai-liang Shi, Song Yang, Yin-long Zhao, Hong-jiang Liu, Chen Li, Hong-lei Liu, Ji-peng Yang, Jian Song, Guo-zhu Sun, Jian-kai Yang

**Affiliations:** 1 Department of Neurosurgery, The Second Hospital of Hebei Medical University, Shijiazhuang, 050000 Hebei China; 2 Medical Department, The First Hospital of Hebei Medical University, Shijiazhuang, 050000 Hebei China; 3 Department of Neurosurgery, Hebei General Hospital, Shijiazhuang, 050000 Hebei China; 4 Department of Anesthesiology and Intensive Care, The Second Hospital of Hebei Medical University, Shijiazhuang, 050000 Hebei China; 5 Department of Neurosurgery, Shijiazhuang Third Hospital, Shijiazhuang, 050011 Hebei China

**Keywords:** Glioma stem cell, E2F4, MCM8, ATR, Enhancer

## Abstract

**Background:**

Glioma stem cells (GSCs) are responsible for glioma recurrence and drug resistance, yet the mechanisms underlying their maintenance remains unclear. This study aimed to identify enhancer-controlled genes involved in GSCs maintenance and elucidate the mechanisms underlying their regulation.

**Methods:**

We analyzed RNA-seq data and H3K27ac ChIP-seq data from GSE119776 to identify differentially expressed genes and enhancers, respectively. Gene Ontology analysis was performed for functional enrichment. Transcription factors were predicted using the Toolkit for Cistrome Data Browser. Prognostic analysis and gene expression correlation was conducted using the Chinese Glioma Genome Atlas (CGGA) data. Two GSC cell lines, GSC-A172 and GSC-U138MG, were isolated from A172 and U138MG cell lines. qRT-PCR was used to detect gene transcription levels. ChIP-qPCR was used to detect H3K27ac of enhancers, and binding of E2F4 to target gene enhancers. Western blot was used to analyze protein levels of p-ATR and γH2AX. Sphere formation, limiting dilution and cell growth assays were used to analyze GSCs growth and self-renewal.

**Results:**

We found that upregulated genes in GSCs were associated with ataxia-telangiectasia-mutated-and-Rad3-related kinase (ATR) pathway activation, and that seven enhancer-controlled genes related to ATR pathway activation (LIN9, MCM8, CEP72, POLA1, DBF4, NDE1, and CDKN2C) were identified. Expression of these genes corresponded to poor prognosis in glioma patients. E2F4 was identified as a transcription factor that regulates enhancer-controlled genes related to the ATR pathway activation, with MCM8 having the highest hazard ratio among genes positively correlated with E2F4 expression. E2F4 bound to *MCM8* enhancers to promote its transcription. Overexpression of MCM8 partially restored the inhibition of GSCs self-renewal, cell growth, and the ATR pathway activation caused by E2F4 knockdown.

**Conclusion:**

Our study demonstrated that E2F4-mediated enhancer activation of *MCM8* promotes the ATR pathway activation and GSCs characteristics. These findings offer promising targets for the development of new therapies for gliomas.

**Supplementary Information:**

The online version contains supplementary material available at 10.1186/s41065-023-00292-x.

## Introduction

Gliomas are common tumors of the central nervous system that can be classified into four grades based on histological features according to the World Health Organization (WHO) [1, 2]. Low-grade gliomas (LGG) are WHO grade I and II gliomas, with less proliferative and invasiveness and better prognosis. High-grade gliomas (HGG) contain WHO grade III and IV gliomas, and generally have a poor prognosis. Although the prognosis for LGG patients is favourable, there is a risk of recurrence and transformation to HGG.

Gliomas are highly heterogeneous tumors that are believed to originate from normal neural stem cells (NSCs) or progenitor cells [3, 4]. Glioma stem cells (GSCs) are an important subpopulation in glioma with self-renewing capability and multidirectional differentiation potential, which are involved in constructing the heterogeneity of glioma microenvironment [5–7]. The clinical significance of GSCs lies in their important contribution on key factors leading to poor prognosis of glioma including radioresistance, chemoresistance, invasion and recurrence [6, 8, 9]. However, the mechanism underlying GSCs maintain the stemness characteristics is still not fully understood. Elucidating the molecular mechanisms that maintain the properties of GSCs is essential for dissecting the development of glioma and improving therapeutic approaches.

Enhancers are segments of DNA sequences with transcriptional regulatory functions that are important for *cis*-regulatory control of gene expression [10]. Enhancers bind to transcription factors through sequence-specific transcription factor recognition and binding sites. Enhancers exist in at least three states including active, primed and poised enhancers [11]. Acetylation of lysine 27 on histone 3 (H3K27ac) is a key hallmark in distinguishing active enhancers from inactive enhancers and this feature is significantly elevated in active enhancers [10]. Poised enhancers are marked by elevated trimethylation at lysine 27 of histone H3 (H3K27me3) [12]. Transcription factors not only play key roles in regulating the activation of enhancers, but also combine with active enhancers to promote transcription of target genes. Active enhancers are considered to be cell specificity. Enhancer-centered regulation is one of the research hotspots for glioma progression. It has been reported that the CREB-binding protein (CBP) and the Bromodomain and Extra-Terminal (BET) inhibitors can reverse the abnormal activation of oncogene enhancers thus inhibiting glioma progression in the H3.3K27M-mutant gliomas [13]. Temozolomide significantly enhances H3K27ac levels at the enhancer of interleukin-8 (IL-8) locus to promote its expression, which in turn IL-8 promotes the acquisition of stem cell properties such as self-renewal and chemoresistance of glioma cells [14]. However, systematic mechanisms by which enhancer-binding transcription factors regulate glioma progression remain a territory that has not been elucidated in detail.

In this study, we demonstrated that function of the upregulated genes in GSCs was associated with the ataxia-telangiectasia-mutated-and-Rad3-related kinase (ATR) pathway activation, and identified enhancer-regulated genes related to the ATR pathway activation. Subsequently, the key transcription factor regulating enhancer-regulated genes associated with the ATR pathway activation was screened and validated. Finally, we explored the impact of the key transcription factor on regulating GSCs traits by affecting the ATR pathway activation. This study attempts to provide a theoretical foundation for the discovery of effective ATR pathway inhibition targets for glioma.

## Materials and methods

### Differential gene identification and functional enrichment

RNA-seq data from the GEO dataset with accession number GSE119776 was utilized to identify differentially expressed genes between GSCs and NSCs. The GSE119776 dataset consists of 44 GSC samples with RNA-seq data and 9 NSC samples with RNA-seq data. The Limma package in R was used to perform differential expression analysis, with cutoff of |log2 fold change|> 1.0 and *P*-value < 0.05.

For functional enrichment, upregulated genes in GSCs were subjected to Gene Ontology (GO) analysis using Metascape (https://www.metascape.org/). The results were then visualized using circos package in R. Enrichment with *P*-value < 0.05 was considered statistically significant.

### Differential H3K27ac signal and enhancer identification

H3K27acChIP-seq data of GSCs (*n* = 43) and NSCs (*n* = 9) were downloaded from GSE119776 for the analysis of H3K27ac signals. The differential H3K27ac signals were identified using the Limma package in R, with a threshold set at |log2 fold change|> 1.0 and P-value < 0.05. The findPeaks package in HOMER was used to analyze the H3K27ac peaks, which were then visualized using the Integrative Genomics Viewer (https://igv.org). Locus enriched with H3K27ac signals were identified as enhancers. Gene closest to an enhancer center was annotated using the annotatePeaks package in HOMER.

### Prognostic analysis

Hazard ratios of genes were calculated based on the transcription profile and clinical data of glioma in the Chinese Glioma Genome Atlas (CGGA; http://www.cgga.org.cn/). Hazard ratios and 95% confidence intervals (CI) were calculated using Cox proportional hazard models. The mRNAseq_693 dataset from the CGGA database was used to analyze the effect of E2F4 and TFDP1 expression on overall survival in all WHO grades, WHO grade II, III and IV primary and recurrent glioma patients. P-value < 0.05 was considered significant.

### Transcription factor prediction

Toolkit for Cistrome Data Browser (http://dbtoolkit.cistrome.org/) was employed to predict transcription factors with default parameters.

### Expression correlation analysis

To determine the expression correlation among genes, the mRNAseq_693 dataset in CGGA database was utilized. A significant positive correlation was defined as a correlation coefficient (R) > 0.4 and *P*-value < 0.05.

### Cell culture

Two glioma cell lines, A172 and U138MG, were purchased from American Type Culture Collection (ATCC, USA). A172 and U138MG cells were cultured in DMEM (Gibco, USA) supplemented with 10% FBS (Gibco, USA) and 1% penicillin–streptomycin (Gibco, USA) at 37 °C in 5% CO_2_. The human neural stem cells (hNSCs; Invitrogen, USA), derived from H9 human embryonic stem cells, were cultured in StemPro NSC SFM medium (Thermo Fisher, USA) at 37 °C in 5% CO_2_.

### GSCs isolation

GSCs were isolated from A172 and U138MG cells according to the previous study [15], by culturing A172 and U138MG cells in the serum-free DMEM (Gibco, USA) supplemented with 2% B27 (Gibco, USA), 20 ng/mL epidermal growth factor (EGF; Gibco, USA) and 20 ng/mL basic fibroblast growth factor (bFGF; Gibco, USA) [16, 17]. The medium was refreshed every three days, and cells were observed every day by an inverted phase-contrast microscope (Leica DMIRB, Italy). Spherical cells after three passages were validated by detecting the expression of the stemness marker CD133 [18] by Western blot. GSCs isolated from A172 and U138MG cells were named GSC-A172 and GSC-U138MG, respectively.

### GSCs transfection

siRNA target E2F4 (si-E2F4), siRNA control (si-ctrl), the MCM8 overexpression plasmid (OE-MCM8) and empty plasmid (OE-ctrl) were purchased from GenePharma (Shanghai, China). Cells were seeded in 6-well plates and allowed to reach 80% confluence before transfection with Lipofectamine 3000 (Invitrogen, USA), following the manufacturer’s instructions.

### Quantitative Real-time PCR (qRT-PCR)

Total RNA was extracted from hNSCs, GSC-A172 and GSC-U138MG cells using TRIzol reagent. cDNA was synthesized from 1 μg of total RNA using the PrimerScript™ RT Reagent Kit (TaKaRa, China). qRT-PCR was performed using the SYBR Premix Ex Taq (TaKaRa, China). RNA expression levels were normalized to the internal reference gene (GAPDH) and quantified using the 2^−ΔΔCt^ method. The sequences of primers for qRT-PCR as following:

DBF4, forward, 5’-GGGCAAAAGAGTTGGTAGTGG-3’; reverse, 5’-ACTTATCGCCATCTGTTTGGATT-3’.

HUS1, forward, 5’-GAATGCCAGGGCTTTGAAAATC-3’; reverse, 5’-CACAATGCGGCTACTGCTTG-3’.

NDE1, forward, 5’-TCTGGCGATGACCTACAAACA-3’; reverse, 5’-CTGCGTCTCCAATTCAGCTT-3’.

POLA1, forward, 5’-AGAAGCTCGCAGTGACAAAAC-3’; reverse, 5’-AGGTGGTGGAGTTATTTGAGGT-3’.

MCM8, forward, 5’-AATGGAGAGTATAGAGGCAGAGG-3’; reverse, 5’-CAGAAGTACGTTTTCCTGTGGT-3’.

E2F4, forward, 5’-CACCACCAAGTTCGTGTCCC-3’; reverse, 5’-GCGTACAGCTAGGGTGTCA-3’.

GAPDH, forward, 5’-GGAGCGAGATCCCTCCAAAAT-3’; reverse, 5’-GGCTGTTGTCATACTTCTCATGG-3’.

### Chromatin immunoprecipitation (ChIP)

Cells were crosslinked with 1% formaldehyde to preserve the protein-DNA interactions, followed by sonication to fragment the chromatin. The resulting chromatin was immunoprecipitated with anti-H3K27ac (ab4729, Abcam, USA), anti-E2F4 (#40,291, Cell Signaling, USA) or anti-IgG (ab171870, Abcam, USA) at 4 °C overnight. The precipitated DNA fragments were purified using a gel extraction kit (QIAGEN, Germany), and subjected to qPCR analysis. Primer sequences for ChIP-qPCR were listed as following:

CDKN2C-E1, forward, 5’-GCATCACTCTCCTTCCTCGG-3’; reverse, 5’- AGGTCGTAACGATTGCCCAG-3’.

CDKN2C-E2, forward, 5’-ACGTCGGGAAACTTGGTCTC-3’; reverse, 5’- GGAAGGCTTGGGTTGGCTAT-3’.

MCM8-E1, forward, 5’-CGTTTCAGCACCACGAAGTC-3’; reverse, 5’- CTCAAAGAAGCGGCAAGACG-3’.

MCM8-E2, forward, 5’-GCGCGGTCATCCTATCTTGT-3’; reverse, 5’- CTTCGCGACGCTTTTACGAC-3’.

### Sphere formation, limiting dilution and cell growth assay

For sphere formation, cells were seeded into 96-well plates at a density of 1000 cells per well. Cell spheres were observed by an inverted phase-contrast microscope (× 400) (Leica DMIRB, Italy), and the diameter of the cell spheres was measured using the ImageJ software on the seventh day post-seeding.

For limiting dilution assay, serial dilutions of the single-cell suspensions were prepared in a 96-well plate (20, 40, 60, 80, 100, 120 or 140 cells per well), with each well containing a final volume of 200 μL complete culture medium. The plates were incubated for two weeks at 37 °C in 5% CO_2_. Extreme Limiting Dilution Analysis (https://bioinf.wehi.edu.au/software/elda/) was used to calculate the sphere formation efficiency.

For cell growth assay, cells were plated in 24-well plates at a density of 1 × 10^5^ cells per well, and incubated for one week at 37 °C in 5% CO_2_. Cells were observed by an inverted phase-contrast microscope (Leica DMIRB, Italy). A hemocytometer was used to count the cell number.

### Western blot

GSCs were lysed using RIPA buffer (Sigma-Aldrich, USA). Protein concentration was determined using a BCA protein assay kit (Thermo Fisher Scientific, USA). Equal amounts of protein (30 μg) were resolved by SDS-PAGE and transferred onto a polyvinylidene difluoride (PVDF) membrane (Millipore, USA). The membrane was blocked with 5% non-fat dry milk for 1 h at room temperature, and then incubated overnight at 4 °C with primary antibodies. Then, the membrane was incubated with HRP Anti-Rabbit IgG antibody (1:5000 dilution, ab288151, Abcam, USA) for 1 h at room temperature. Proteins were visualized using an ECL detection system (Thermo Fisher Scientific, USA), and the intensity of the target band was quantified using ImageJ software. The primary antibodies were listed as follows: anti-CD133 (1:5000 dilution, ab19898, abcam, USA), anti-p-ATR (phospho T1989) (1:5000 dilution, ab223258, abcam, USA), anti-γH2AX (1:5000 dilution, ab81299, abcam, USA) and anti-GAPDH (1:5000 dilution, ab8245, abcam, USA).

### Statistical analysis

All data are expressed as the mean ± SEM. Statistical analysis was performed using Student’s *t*-test or one-way analysis of variance followed by Tukey’s post-hoc test. P-value of less than 0.05 was considered statistically significant.

## Results

### Upregulated genes in GSCs were associated with activation of ATR pathway

**Fig. 1:**
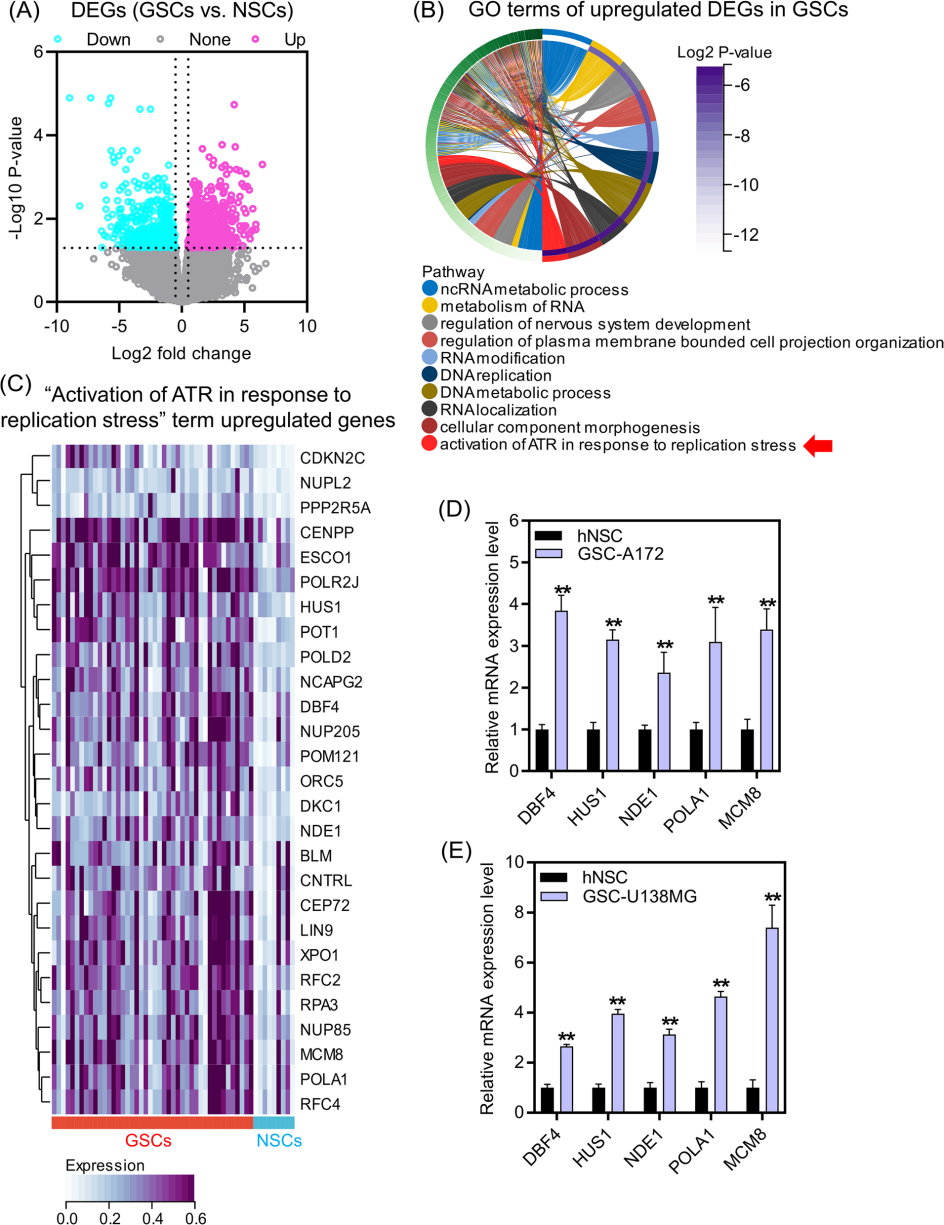
Upregulated genes in GSCs were related to ATR pathway activation. **A** Differential gene expression analysis was performed between GSCs and NSCs based on GSE119776 dataset. Differentially expressed genes were filtered with |log2 fold change|≥ 1.0 and *P*-value < 0.05. **B** The top ten GO terms of upregulated genes in GSCs. A *P*-value < 0.05 indicates significant enrichment. **C** Heatmap of the 27 upregulated genes clustered in the “activation of ATR in response to replication stress” term. (D/E) qRT-PCR analysis was performed to determine the expression levels of DBF4, HUS1, NDE1, POLA1 and MCM8 in hNSCs, GSC-A172 and GSC-U138MG cells. **P-value < 0.01 by Student’s *t*-test, vs. hNSCs

GSCs play a crucial role in promoting glioma progression. To elucidate the mechanisms regulating the GSCs traits, we conducted gene expression comparison between GSCs and NSCs. Our analysis revealed that 596 genes were significantly upregulated, while 432 genes were significantly downregulated in GSCs as compared to NSCs (Fig. 1A). Then, we conducted GO analysis to gain insights into the function of upregulated genes in GSCs. As depicted in (Fig. 1B), the top ten GO terms revealed that the upregulated genes were closely linked to RNA and DNA metabolism, RNA modification and localization, regulation of nervous system development, and cellular component morphogenesis. Intriguingly, “activation of ATR in response to replication stress” was significantly enriched (Fig. 1B). ATR pathway plays important roles in the regulation of various processes such as apoptosis, DNA damage repair and drug resistance in glioma [19–21]. There were twenty-seven genes with elevated expression in GSCs were clustered in the “activation of ATR in response to replication stress” term (Fig. 1C). To validate this finding, we selected five genes (DBF4, HUS1, NDE1, POLA1, and MCM8) out of the twenty-seven genes for qRT-PCR verification, based on their known roles in the ATR signaling and DNA damage responses [22–26]. The expression of the five genes was obviously enhanced in GSC-A172 and GSC-U138MG cells compared to hNSCs (Fig. 1D/E). In summary, our study highlighted the enrichment of the upregulated genes in GSCs compared to hNSCs in the “activation of ATR in response to replication stress” term.

### Enhancer-controlled genes are associated with ATR pathway activation in GSCs

**Fig. 2:**
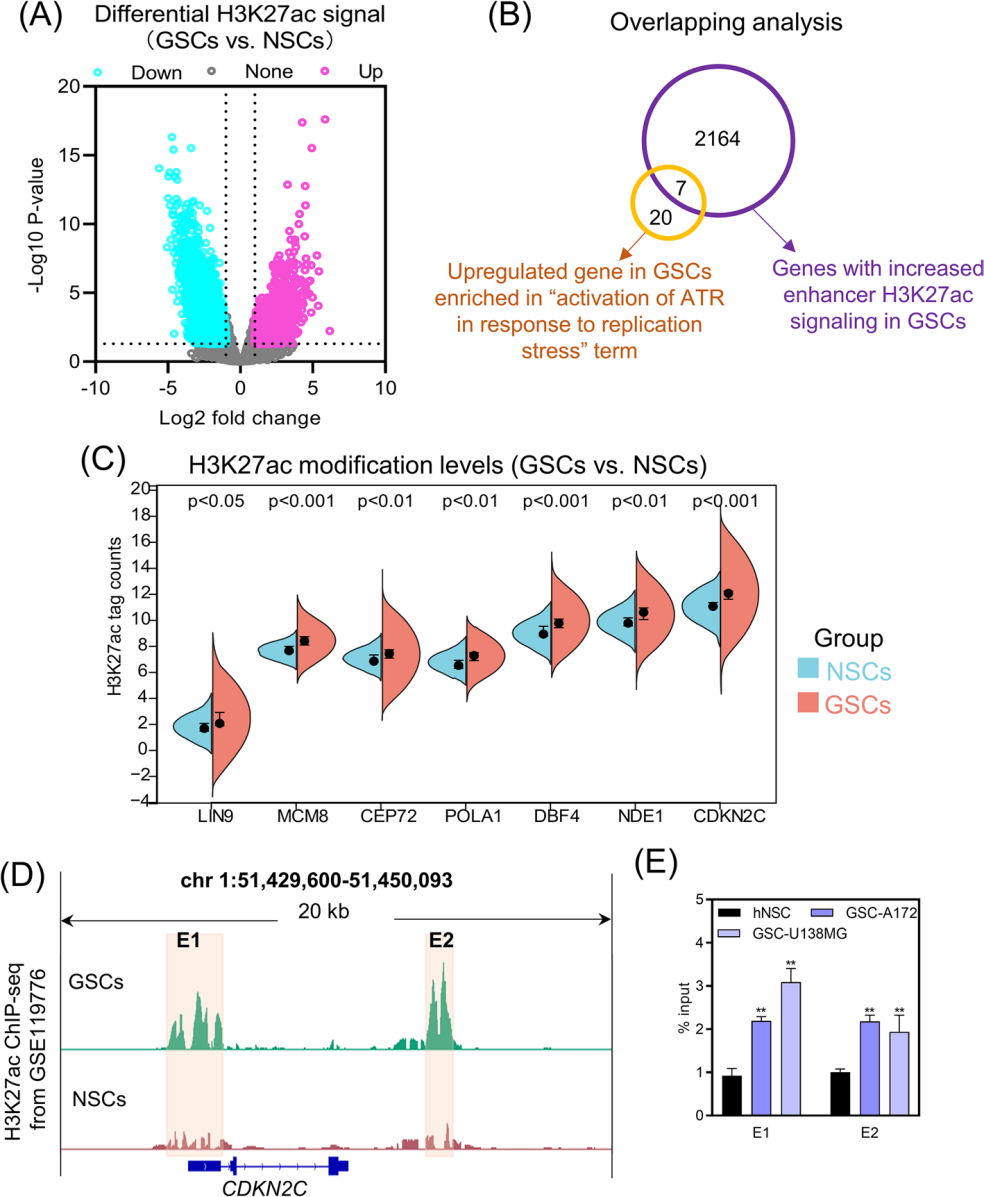
Characterization of enhancer-controlled genes in “activation of ATR in response to replication stress” term. **A** Differential analysis of H3K27ac signal in GSCs vs. NSCs based on GSE119776 dataset. The cutoff value was |log2 fold change|≥ 1.0 and *P*-value < 0.05. **B** Venn diagram showing the intersection of genes with increased enhancer H3K27ac signaling (purple) and upregulated gene enriched in “activation of ATR in response to replication stress” term (yellow). **C** Comparison of H3K27ac modification levels at the *LIN9*, *MCM8*, *CEP72*, *POLA1*, *DBF4*, *NDE1* and *CDKN2C* loci in GSCs vs. NSCs. Statistical analysis was performed using Student’s *t*-test. **D** Distribution of H3K27ac peaks at the *CDKN2C* locus was determined using GSE119776 dataset. Regions with significantly elevated H3K27ac signal in GSCs were labelled as region E1 and E2. **E** ChIP-qPCR analysis of anti-H3K27ac enrichment of region E1 and E2. **P-value < 0.01 by one-way analysis of variance, vs. hNSCs

To further investigate the regulatory mechanisms of genes associated with ATR pathway activation, we analyzed the differences in H3K27ac modifications between GSCs and NSCs using GSE119776 dataset. A total of 16,747 H3K27ac signals were significantly different between GSCs and NSCs, of which 5726 signals were significantly elevated in GSCs (Fig. 2A). Enhancers were annotated via enrichment of H3K27ac signals. Genes nearest to the center of enhancer regions on the genome were assigned as enhancer-controlled genes. In the present study, 2171 genes were identified as genes with elevated enhancer H3K27ac signaling in GSCs (Fig. 2B). By intersecting the genes with increased enhancer H3K27ac signaling and the upregulated gene enriched in the “activation of ATR in response to replication stress” term, we identified seven genes, including LIN9, MCM8, CEP72, POLA1, DBF4, NDE1 and CDKN2C (Fig. 2B). The H3K27ac tag counts of the seven intersecting genes were markedly higher in GSCs than in NSCs (Fig. 2C). CDKN2C was chosen for validation of the H3K27ac modification as it displaying the highest H3K27ac tag counts (Fig. 2C). The enhancers of *CDKN2C* were divided into two regions (E1 and E2) based on the intensity of the H3K27ac signal (Fig. 2D). H3K27ac signal at the region E1 and E2 of the *CDKN2C* locus was significantly higher in GSCs than NSCs (Fig. 2D). Subsequently, we used ChIP-qPCR to detect the levels of H3K27ac in region E1 and E2. As expected, the enrichment of anti-H3K27ac in region E1 and E2 of GSC-A172 and GSC-U138MG cells was significant compared to hNSCs (Fig. 2E). In general, LIN9, MCM8, CEP72, POLA1, DBF4, NDE1 and CDKN2C emerged to be the genes controlled by enhancers enriched in the “activation of ATR in response to replication stress” term.

### Enhancer-controlled genes associated with ATR pathway activation increase the prognostic risk in glioma patients

**Fig. 3:**
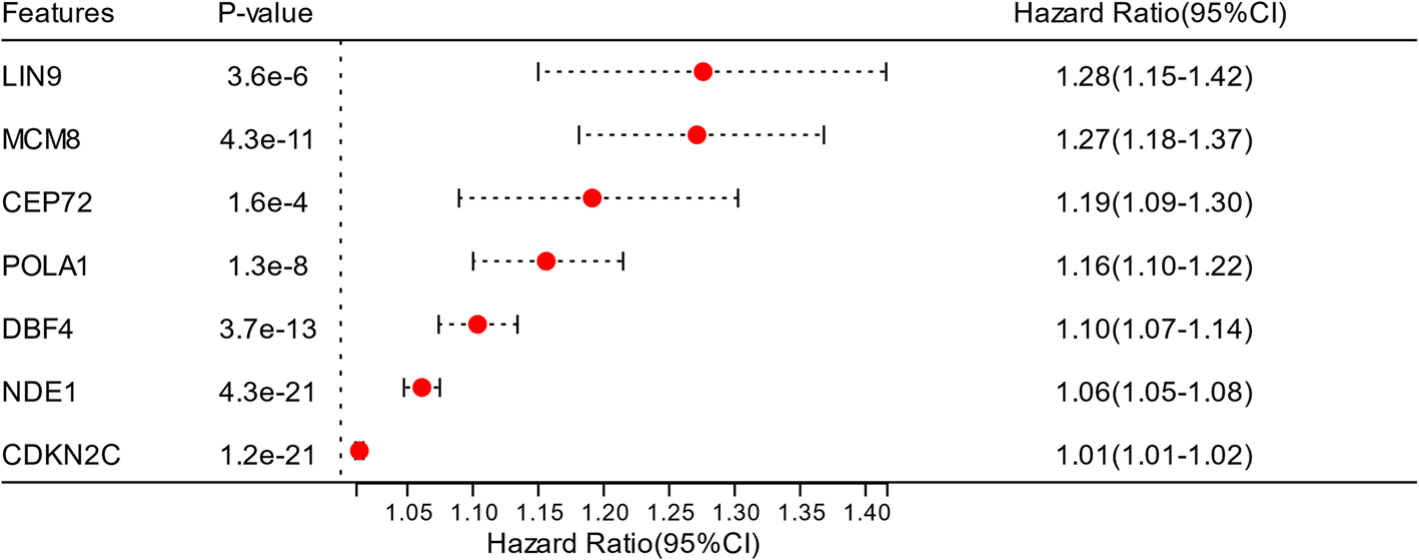
Forest plots of hazard ratios and 95% confidence intervals (CI) for the overall survival of glioma patients of the genes LIN9, MCM8, CEP72, POLA1, DBF4, NDE1 and CDKN2C

To evaluate the prognostic risk related to the enhancer-controlled genes associated with ATR pathway activation, we calculated the hazard ratio and 95% confidence interval (CI) of the overall survival of glioma patients using Cox proportional hazard models. The seven investigating genes have a hazard ratio greater than one and are ranked in descending order of hazard ratio: LIN9 (hazard ratio = 1.28), MCM8 (hazard ratio = 1.27), CEP72 (hazard ratio = 1.19), POLA1 (hazard ratio = 1.16), DBF4 (hazard ratio = 1.10), NDE1 (hazard ratio = 1.06) and CDKN2C (hazard ratio = 1.01) (Fig. 3). Overall, the expression of LIN9, MCM8, CEP72, POLA1, DBF4, NDE1 and CDKN2C increase the prognostic risk for patients with glioma.

### E2F4 was screened as the transcription factor regulating enhancer-controlled genes clustered in “activation of ATR in response to replication stress” term

**Fig. 4:**
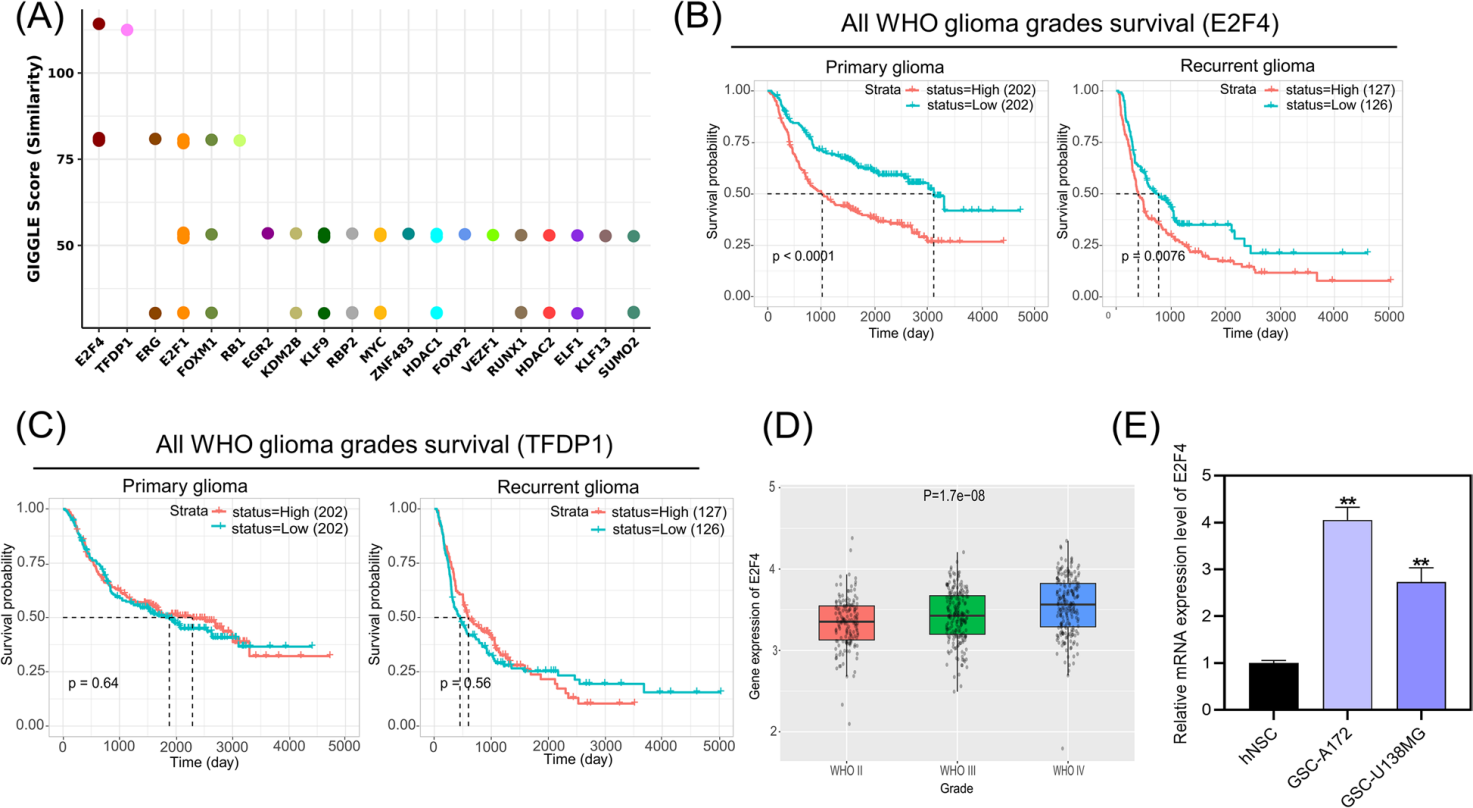
Prediction and clinical characterization of transcription factors regulating enhancer-controlled genes associated with ATR pathway activation. **A** Potential transcription factors for the genes LIN9, MCM8, CEP72, POLA1, DBF4, NDE1 and CDKN2C were ranked by the GIGGLE scores. **B/C** Overall survival of all WHO grades primary and recurrent glioma patients was analyzed based on the expression of E2F4 (**B**) and TFDP1 (**C**), which were identified as the potential transcription factors. Patients were grouped into high and low groups according to the median expression levels. The data were obtained from the Chinese Glioma Genome Atlas (CGGA) database. **D** Differential analysis of E2F4 was performed in different WHO glioma grades. Statistical significance was analyzed using one-way analysis of variance. **E** The expression of E2F4 was analyzed using qRT-PCR in hNSCs, GSC-A172 and GSC-U138MG cells. **P-value < 0.01 by Student’s *t*-test, vs. hNSCs

To further explore the mechanism underlying the expression of enhancer-controlled genes clustered in “activation of ATR in response to replication stress” term, we predicted the transcription factors of LIN9, MCM8, CEP72, POLA1, DBF4, NDE1 and CDKN2C using the Toolkit for Cistrome Data Browser. The potential transcription factors were arranged in descending order by GIGGLE scores, and the top twenty were displayed in Fig. 4A. The top two transcription factors, E2F4 and TFDP1, were selected for prognostic analysis using CGGA. The expression of E2F4, but not TFDP1, is associated with poor overall survival of all WHO grades in both primary and recurrent glioma patients (Fig. 4B/C). E2F4 expression has no significant effect on overall survival in patients with WHO grade II glioma or WHO grade IV recurrent glioma (Fig. S1A, B and F). Patients with WHO grade III glioma or WHO grade IV primary glioma with high E2F4 expression have a poor overall survival (Fig. S1C, D and E). TFDP1 expression has no significant effect on the overall survival of patients with WHO grade II, III and IV primary and recurrent gliomas (Fig. S2). Moreover, E2F4 expression is increasing with the WHO glioma grade (Fig. 4D).

The correlation between E2F4 and LIN9, MCM8, CEP72, POLA1, DBF4, NDE1 or CDKN2C expression in glioma tissues was analyzed using CGGA. A significant positive correlation was defined as R > 0.4 and *P*-value < 0.05. Our analysis revealed a significant positive correlation between E2F4 and MCM8, POLA1, DBF4, NDE1 or CDKN2C expression in primary gliomas (Table 1). In recurrent gliomas, E2F4 showed a significant positive correlation with the expression of MCM8, DBF4 and CDKN2C (Table 1). The expression of MCM8, DBF4 and CDKN2C displayed a significant positive correlation with E2F4 expression in both primary and recurrent glioma tissues (Table 1).

**Table 1:** Pearson’s correlation coefficients of E2F4 and LIN9, MCM8, CEP72, POLA1, DBF4, NDE1 or CDKN2C expression in glioma tissues using the Chinese Glioma Genome Atlas (CGGA) database

Gene	Primary gliomas		Recurrent gliomas	
	R	*P*-value	R	*P*-value
LIN9	0.382	1.31E-12	0.333	7.50E-08
MCM8	0.489	9.02E-21	0.403	4.41E-11
CEP72	0.273	6.47E-07	0.332	8.59E-08
POLA1	0.474	1.83E-19	0.364	3.31E-09
DBF4	0.513	5.09E-23	0.49	2.32E-16
NDE1	0.476	1.33E-19	0.338	4.79E-08
CDKN2C	0.553	3.3E-27	0.403	4.03E-11

Furthermore, we conducted qRT-PCR analysis to measure E2F4 expression. The results showed that E2F4 was significantly upregulated in GSC-A172 and GSC-U138MG cells compared to hNSCs (Fig. 4E). Taken together, these findings suggested that E2F4 may serve as a transcription factor regulating enhancer-controlled genes clustered in the “activation of ATR in response to replication stress” term.

### E2F4 combined with the enhancer of MCM8 to promote MCM8 transcription

**Fig. 5:**
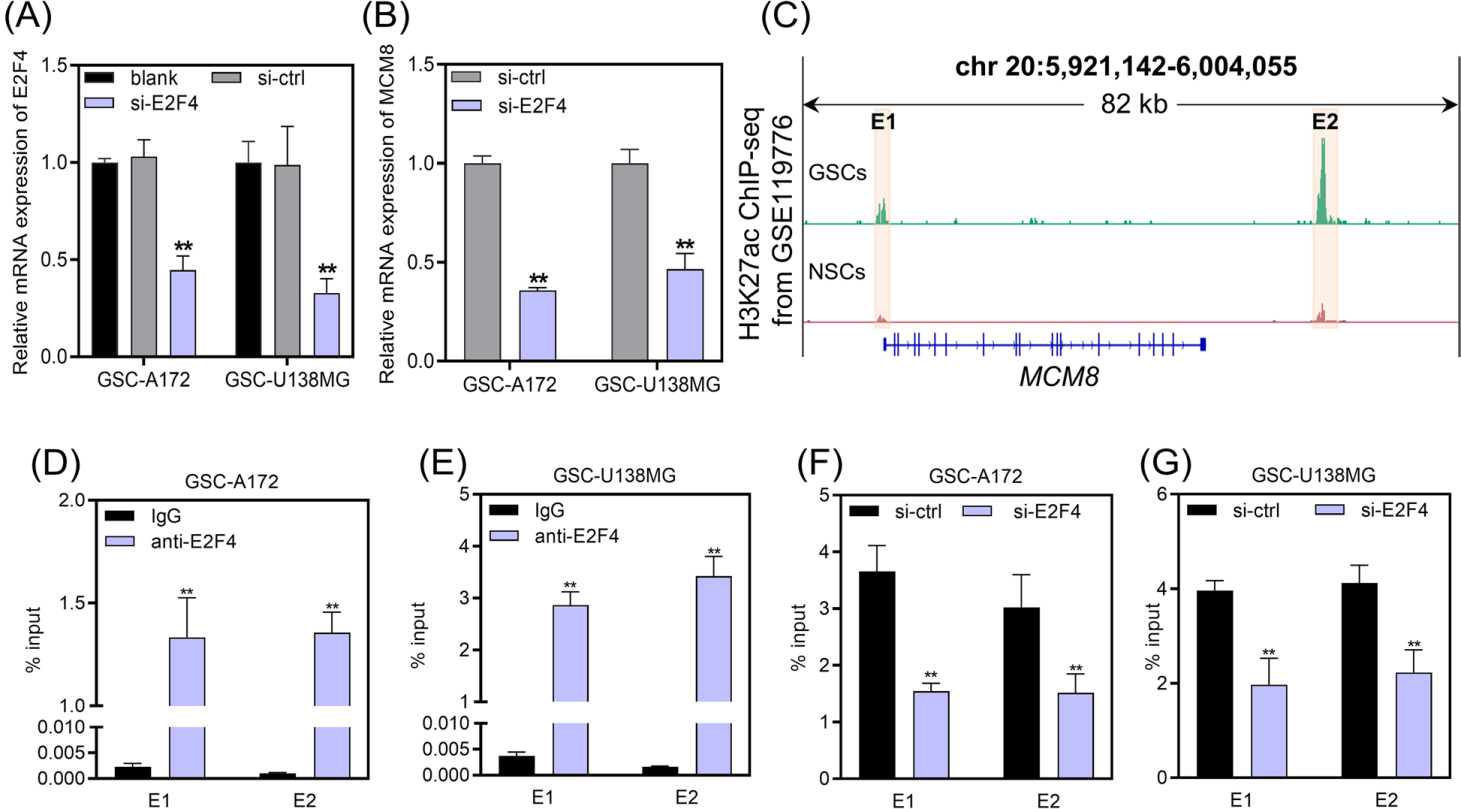
E2F4 promoted *MCM8* transcription by binding to its enhancer. **A** qRT-PCR analysis was used to measure E2F4 expression in GSCs transfected with si-ctrl or si-E2F4. **P-value < 0.01 by one-way analysis of variance, vs. si-ctrl. **B** qRT-PCR analysis was used to measure MCM8 expression in GSCs transfected with si-ctrl or si-E2F4. **P-value < 0.01 by Student’s *t*-test, vs. si-ctrl. **C** H3K27ac peaks at the *MCM8* locus of GSCs and NSCs. The upregulated enhancer regions in GSCs compared to NSCs were marked as region E1 and E2. **D/E** ChIP-qPCR analysis of anti-E2F4 enrichment at region E1 and E2 in GSCs. **P-value < 0.01 by Student’s *t*-test, vs. IgG group. **F/G** ChIP-qPCR was used to detect the enrichment of anti-H3K27ac on the enhancer regions of *MCM8* in GSCs transfected with si-ctrl or si-E2F4. **P-value < 0.01 by Student’s *t*-test, vs. si-ctrl group

Among the genes (MCM8, DBF4 and CDKN2C) that showed a significant positive correlation with E2F4 expression in both primary and recurrent tissues (Table 1), MCM8 displayed the highest hazard ratio (hazard ratio = 1.27 for MCM8, hazard ratio = 1.10 for DBF4, and hazard ratio = 1.01 for CDKN2C) (Fig. 3), making it the focus of this study. For that reason, we wanted to further investigate the effect of E2F4 on MCM8 transcription. To achieve this, we knocked down E2F4 in two GSC cell lines (GSC-A172 and GSC-U138MG), which were confirmed by qRT-PCR (Fig. 5A). Transfection of si-E2F4 significantly inhibited MCM8 expression (Fig. 5B).

Then, we analyzed H3K27ac peaks at the *MCM8* locus using GSE119776 dataset, and identified the enhancer regions (region E1 and E2) of *MCM8* that were upregulated in GSCs compared to NSCs (Fig. 5C). The binding of E2F4 to *MCM8* enhancer regions in GSC-A172 and GSC-U138MG cells was demonstrated by ChIP-qPCR (Fig. 5D/E). We used ChIP-qPCR to detect the effect of E2F4 knockdown on the H3K27ac levels of *MCM8* enhancer regions. Knockdown of E2F4 resulted in a significant downregulation of H3K27ac levels of *MCM8* enhancer regions in GSCs (Fig. 5F/G). The above results indicated that transcription factor E2F4 interacts with the enhancer of *MCM8* and promotes its transcription.

### MCM8 overexpression restored the inhibition of GSCs growth, self-renewal and ATR activation induced by E2F4 knockdown

**Fig. 6:**
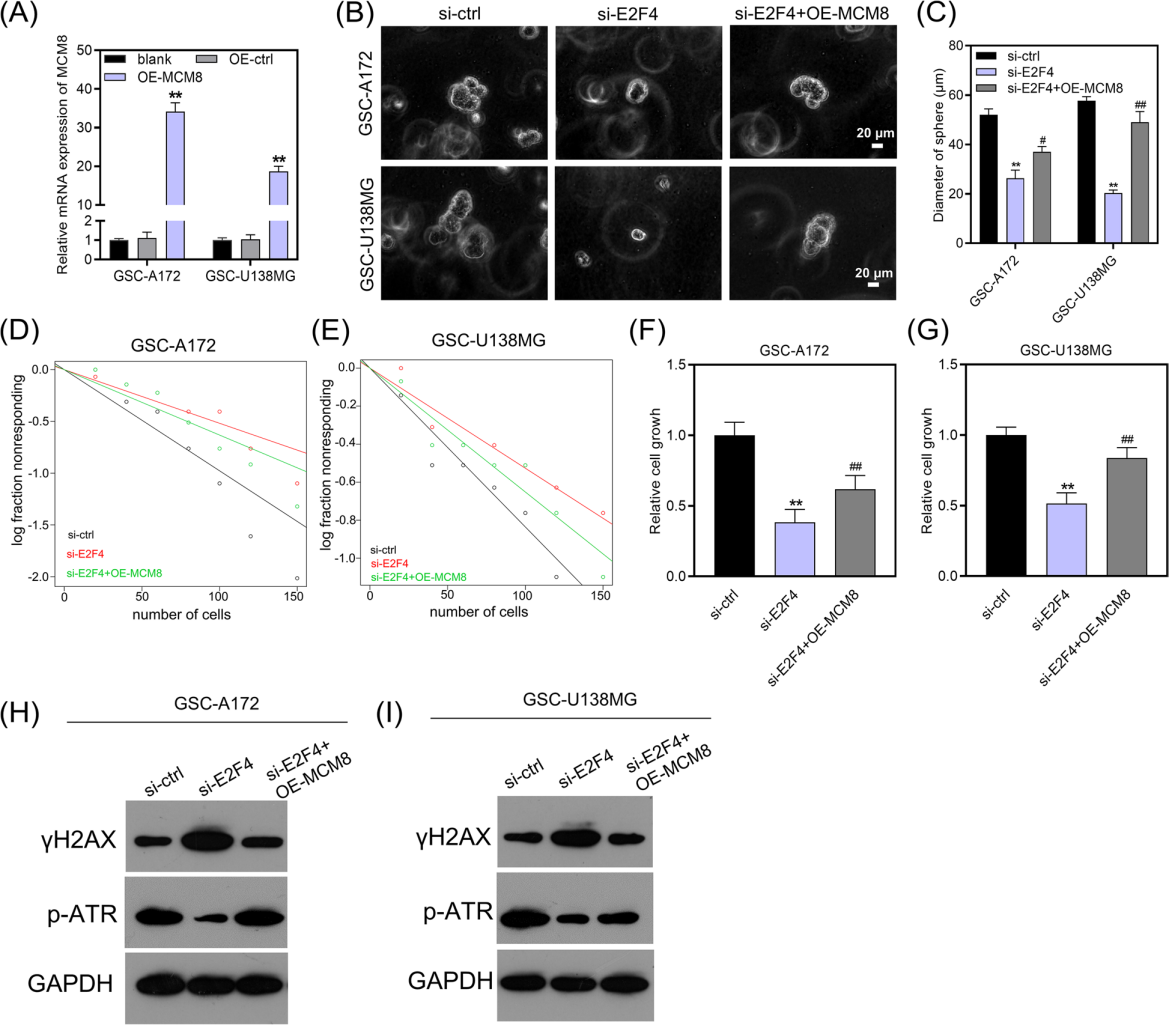
MCM8 overexpression counteracted the effects of E2F4 knockdown on GSCs traits. **A** qRT-PCR analysis was used to measure MCM8 expression in OE-ctrl and OE-MCM8 groups. **P-value < 0.01 by one-way analysis of variance, vs. OE-ctrl. **B/C** Tumor sphere formation of GSCs transfected with si-ctrl, si-E2F4 or co-transfected with si-E2F4 and OE-MCM8 plasmid. **P-value < 0.01 by one-way analysis of variance, vs. si-ctrl. ##*P*-value < 0.01 and #*P*-value < 0.05 by one-way analysis of variance, vs. si-E2F4. **D/E** Limiting dilution assay of GSCs transfected with si-ctrl, si-E2F4 or co-transfected with si-E2F4 and OE-MCM8 plasmid. **F/G** Relative cell growth of GSCs transfected with si-ctrl, si-E2F4 or co-transfected with si-E2F4 and OE-MCM8 plasmid. **P-value < 0.01 by one-way analysis of variance, vs. si-ctrl. ##*P*-value < 0.01 by one-way analysis of variance, vs. si-E2F4. **H/I** Western blot of p-ATR and γH2AX in GSCs transfected with si-ctrl, si-E2F4 or co-transfected with si-E2F4 and OE-MCM8 plasmid

In order to gain insights into whether E2F4 and MCM8 affect the GSCs characteristics, we examined the effects of E2F4 knockdown and MCM8 overexpression on GSCs cell growth, self-renewal and ATR pathway activation. We verified the efficiency of MCM8 overexpression by qRT-PCR (Fig. 6A). Knockdown of E2F4 hindered the self-renewal ability of GSCs, as evidenced by the reduction in sphere diameter (Fig. 6B/C) and stem cell frequency (Fig. 6D/E) after knockdown of E2F4. However, co-transfection of si-E2F4 and OE-MCM8 plasmid increased sphere diameter and stem cell frequency compared to transfection of si-E2F4 alone, indicating that overexpression of MCM8 counteracted the self-renewal capacity impaired by E2F4 knockdown (Fig. 6B-E). In addition, E2F4 knockdown substantially reduced GSCs cell growth, which was partially restored by overexpression of MCM8 (Fig. 6F/G).

To dissect the roles of MCM8 and E2F4 in the activation of ATR pathway, we examined the protein levels of the phosphorylated form of the ATR activation marker (p-ATR) and the DNA damage marker (γH2AX) upon E2F4 knockdown and MCM8 overexpression. Our results showed that E2F4 knockdown upregulated γH2AX expression, but downregulated p-ATR levels, which could be partially rescued by overexpression of MCM8 (Fig. 6H/I). Together, these results corroborated that E2F4 knockdown inhibited GSCs cell growth, self-renewal and ATR pathway activation, which could be counteracted by MCM8 overexpression.

## Discussion

GSCs exhibit a potent tumor initiation capability and play a dominant role in the malignant progression of gliomas, such as recurrence, chemo- and radio-resistance of gliomas [27–29]. Nonetheless, the molecular mechanism underlying the characteristics of GSCs is still elusive. Our data pinpoint the E2F4 as transcription factor that regulates enhancer-controlled genes related to ATR activation (LIN9, MCM8, CEP72, POLA1, DBF4, NDE1 and CDKN2C). Furthermore, we have demonstrated that E2F4 promotes MCM8 transcription by binding to its enhancer, thereby regulating the GSCs traits by influencing ATR activation.

In this study, we compared the transcription profiles of GSCs and NSCs, and identified 596 genes were upregulated in GSCs, among which 27 genes were enriched in the “activation of ATR in response to replication stress” term. Ataxia-telangiectasia-mutated-and-Rad3-related kinase (ATR) is a vital cell cycle checkpoint protein [30]. When DNA replication stress or damage occurs, ATR is recruited to the damaged site and activated by multiple regulatory proteins, leading to cell cycle arrest, DNA damage repair and other biological processes [31, 32]. Activation of ATR pathway is crucial for maintaining genomic stability and promoting cell survival [33]. Notably, activation of ATR pathway significantly promotes tumor cell survival, while having minimal impact on normal cells [34]. ATR pathway plays a pivotal role in glioma progression. For example, Myc targeted CDK18 affects the interaction of ATR-RAD9 and ATR-ETAA1, and promotes ATR activation, thus promoting homologous recombination and PARP inhibitor resistance in glioma [19]. Silencing ACTL6A can induce glioma cell apoptosis by inhibiting the ATR/CHK1 pathway [20]. NUSAP1 enhances ATR stability by promoting its sumoylation and inhibiting its ubiquitin-dependent degradation, thereby promoting resistance to temozolomide and doxorubicin in glioma [21]. Further elucidation of more detailed ATR pathway-related regulatory mechanisms in glioma needs to be explored.

Abnormal epigenetic regulation can induce various cancers. Enhancer abnormally activation, as an important pathway of epigenetic regulation, is a key mechanism leading to increased gene expression. In recent years, the mechanisms of enhancer regulation in glioma progression have received considerable attention. For example, research has found that in the H3.3K27M-mutant glioma cells, PRC2 complex is sequestered on poised enhancers, which leads to the silencing of tumor suppressor genes by enhancing H3K27me3 modification on their loci [35]. Changes in the activity of an enhancer on 20q13.33 led to abnormal expression of multiple genes associated with glioma risk (e.g., RTEL1, RTEL1-TNFRSF6B, SRMS and GMEB2) [36]. Enhancers have different existence states. Research has confirmed that enhancers can transform into each other among at least three states (active, primed and poised states), among them, those with transcription activation functions are active enhancers [11]. Active enhancers have typical local chromatin marks, exhibiting high levels of H3K27ac deposition [10]. In glioma, the expression of IL-8 is promoted by upregulating H3K27ac at the enhancer locus of the IL-8 gene, which is beneficial for maintaining the GSCs characteristics [14]. However, a more detailed understanding of enhancer regulation of glioma progression, especially the mechanism underlying GSCs traits, is still unclear. In this study, we aimed to determine which genes among those enriched in the “activation of ATR in response to replication stress” term were regulated by enhancers. Analysis of H3K27ac levels across the genome is a viable strategy for identifying active enhancers [37]. Thus, we analyzed H3K27ac signals at the genome-wide level in GSCs and NSCs, and identified 5726 upregulated signals.

The regulation of target genes by enhancers is uncertain in direction and location [38, 39]. In this study, the gene closest to the center of an enhancer was considered to be an enhancer-controlled gene. We identified seven enhancer-controlled genes related to the ATR pathway activation (LIN9, MCM8, CEP72, POLA1, DBF4, NDE1 and CDKN2C). Notably, the expression of these genes increased the prognostic risk in glioma patients, suggesting their potential clinical significance and value for further research.

The promotion of target gene transcription by enhancers is a result of the collaboration of enhancers and transcription factors [40, 41]. The mechanism by which enhancers regulate the stem cell characteristics of GSCs through interacting with transcription factors remains unclear. In this study, E2F4 was selected as a transcription factor regulating the enhancer-controlled genes related to ATR pathway activation. E2F transcription factor 4 (E2F4) is a member of the E2F family and plays a pro- or anti-tumorigenic role in a variety of cancers. E2F4 represses the MAPK signaling pathway by binding with EZH2, thereby inhibiting the progression of acute myeloid leukemia [42]. High expression of E2F4 is an unfavorable prognostic factor in oral squamous cell carcinoma [43]. E2F4 accelerates the progression of colorectal cancer by promoting AGAP2-AS1 expression [44]. In hepatocellular carcinoma, E2F4 promotes the proliferation, migration and invasion of hepatocellular carcinoma cells by upregulating CDCA3 [45]. The role and mechanism of E2F4 in glioma are still not well understood. It has been reported that E2F4 expression is upregulated in glioblastoma cells [46]. Knockdown of E2F4 reduces the levels of proneural markers, but upregulates the levels of mesenchymal markers in proneural GSCs [47]. At present, there are few studies on the regulation of GSCs traits by E2F4. We found that E2F4 expression in GSCs was significantly higher than that in NSCs, and it was identified as an unfavorable prognostic factor for glioma patients. Furthermore, the higher WHO glioma grading, the higher the expression of E2F4, suggesting that E2F4 might play a role in the malignant progression of gliomas.

There was a strong positive correlation between E2F4 expression and the expression of MCM8, DBF4 and CDKN2C in both primary and recurrent glioma tissues, with the most prominent impact on patient prognosis being observed for MCM8. Subsequently, we demonstrated that E2F4 promotes *MCM8* transcription by binding to its enhancer. Minichromosome maintenance 8 (MCM8), a homologous recombination repair factor, plays a crucial role in regulating the initiation and extension of DNA replication [26]. MCM8 contributes to the progression of various cancers, such as cholangiocarcinoma, osteosarcoma, gastric cancer and glioma [48–51]. Abnormal upregulation of MCM8 expression is observed in glioma, and high expression of MCM8 is associated with poor prognosis [51]. MCM8 is regulated by the EGFR signaling, and promotes the growth and tumorigenicity of GSC through its interaction with DNA replication initiation factors [52]. In this study, we demonstrated that overexpression of MCM8 rescues the decline of self-renewal and cell growth induced by E2F4 knockdown.

Given the enrichment of MCM8 in the “activation of ATR in response to replication stress” term, we sought to investigate the impact of aberrant MCM8 and E2F4 expression on the ATR pathway activation in the present study. ATR pathway is essential for maintaining the genomic stability and integrity and thus cell survival [33]. When DNA replication stress or DNA damage occurs, the ATR is recruited to the DNA damage site and subsequently activated to form the p-ATR, which promotes DNA damage repair [53, 54]. Phosphorylation at T1989 is crucial for the activation of ATR, making p-ATR a marker of the ATR pathway activation [55]. γH2AX is the tag for DNA double-strand breaks (DSBs) [56]. Following the appearance of DSBs, H2AX rapidly becomes phosphorylated to form γH2AX and clusters at DSB sites to form foci. γH2AX foci are quantitatively the same as DSBs [57]. High levels of γH2AX represent a great accumulation of DSBs and a poor ability of the cell to repair DSBs. Therefore, we evaluated the levels of p-ATR and γH2AX in this study. In the present study, silencing of E2F4 led to a decrease in p-ATR levels, suggesting a reduction in the ability of DNA damage repair in GSCs. In addition, silencing of E2F4 led to an upregulation of γH2AX levels, suggesting that the accumulation of DSBs and the decreased ability of DNA damage repair in GSCs. Considered from this perspective, the decrease in p-ATR levels and the increase in γH2AX levels following silencing of E2F4 both demonstrated the diminished ability of DNA damage repair and the ATR pathway activation in GSCs. Overexpression of MCM8 partially counteracted the decrease in p-ATR levels and increase in γH2AX levels induced by E2F4 knockdown in GSCs. ATR is frequently expressed at high levels in cancer stem cells playing a crucial role in maintaining their stemness [58]. For instance, knockdown of ATR impairs the stemness characteristics of colon cancer cells, suppressing their tumorigenic ability [59]. These results suggest that E2F4 controls the upregulation of MCM8 transcription, which in turn promotes cell growth and self-renewal of GSCs via activation of ATR pathway. The roles and mechanisms of E2F4 and MCM8 in maintaining the characteristics of GSCs and regulating gliomas progression still needs to be further explored at the in vivo level.

## Conclusion

In summary, this present study suggested that E2F4 promoted *MCM8* transcription by binding to its enhancer, which in turn activated the ATR pathway to promote the growth and self-renewal of GSCs. The findings presented herein indicated that E2F4 and MCM8 represent promising therapeutic targets for the treatment of gliomas.

## Supplementary Information

**Additional file 1.** 

**Additional file 2.** 

## Data Availability

The data of this study are available upon reasonable request by contacting the corresponding author.
