# Supplementary figures and images for: Enhancer-driven transcription of MCM8 by E2F4 promotes ATR pathway activation and glioma stem cell characteristics
Source: Hereditas. 2023 Jun 22;160:29. doi: 10.1186/s41065-023-00292-x (PMC10286446; doi:10.1186/s41065-023-00292-x)

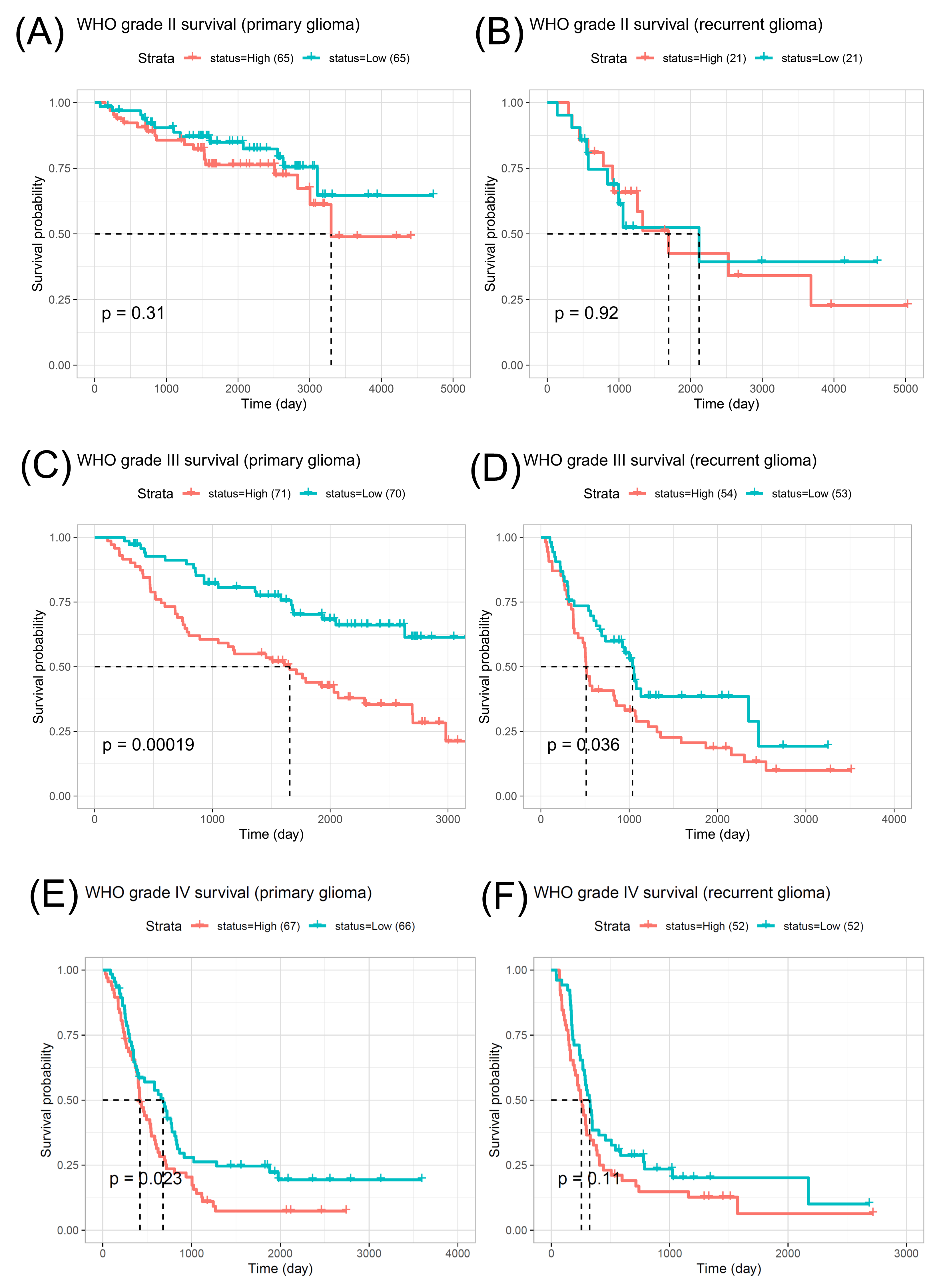

Supplement: Supplementary file 1 — Additional file 1. [file 41065_2023_292_MOESM1_ESM.tif]

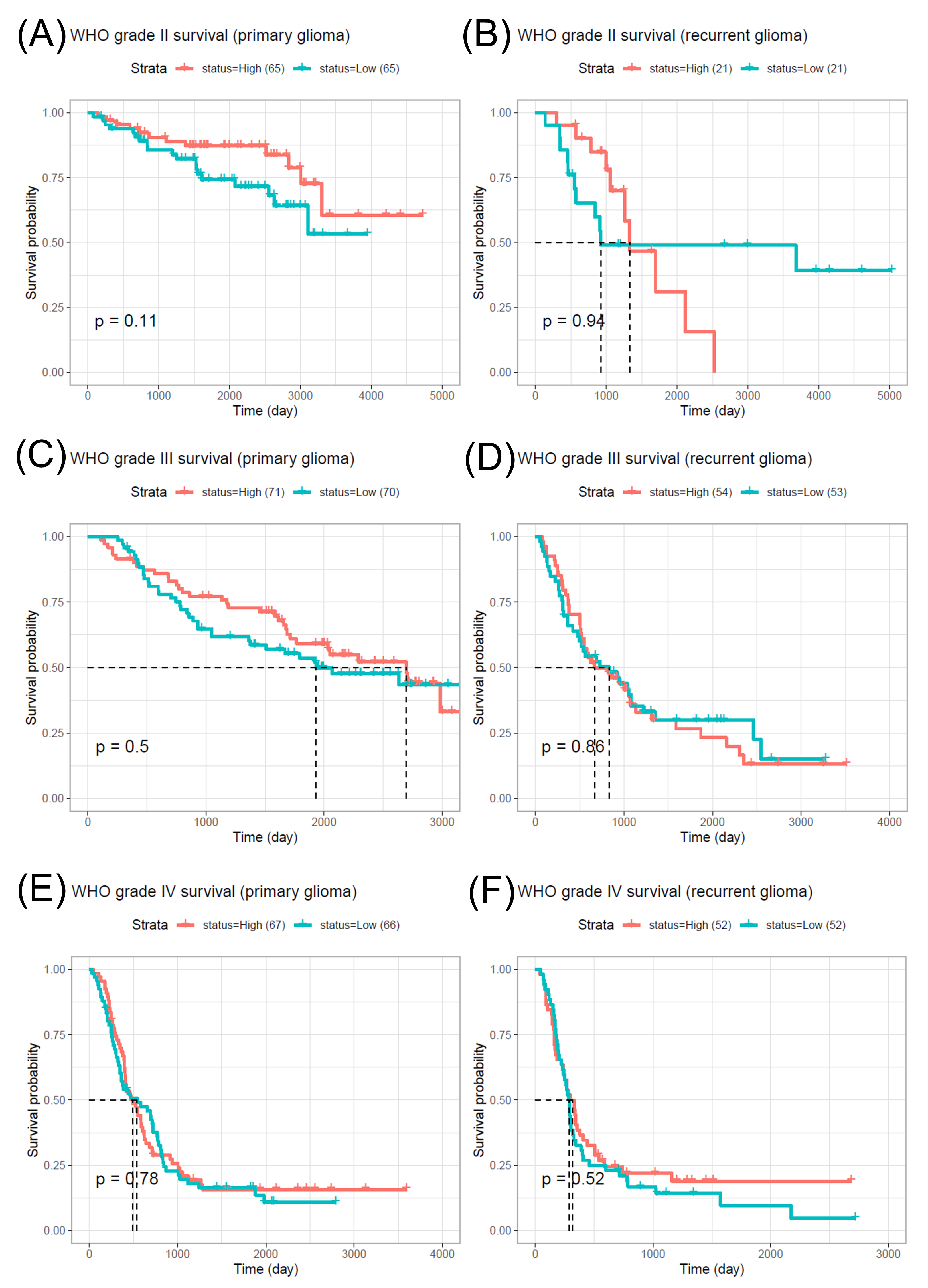

Supplement: Supplementary file 2 — Additional file 2. [file 41065_2023_292_MOESM2_ESM.tif]
